# Single-cell RNA sequencing reveals the therapeutic mechanism of Calvatia lilacina in promoting wound healing of anal fistula

**DOI:** 10.1186/s13020-025-01293-w

**Published:** 2026-01-07

**Authors:** Tangtang He, Kewei Wang, Ruiwen Mo, Juntong Guo, Bin Jiang, Ruoyu Mu, Wen Min, Lifeng Zhu, Jun Chen

**Affiliations:** 1 Jiangsu Provincial Engineering Research Center of Traditional Chinese Medicine External Medication Development and Application, Nanjing University of Chinese Medicine, Nanjing, 210023 China; 2 School of Pharmacy, Nanjing University of Chinese Medicine, Nanjing, 210023 China; 3 Nanjing Hospital of Chinese Medicine Affiliated to Nanjing University of Chinese Medicine, Nanjing, 210022 China; 4 Department of Bone Injury of Traditional Chinese Medicine, Affiliated Hospital of Nanjing University of Chinese Medicine, Nanjing, 210029 China; 5 Key Laboratory of Drug Target and Drug for Degenerative Disease, Nanjing University of Chinese Medicine, Nanjing, 210023 China; 6 School of Medicine, Nanjing University of Chinese Medicine, Nanjing, 210023 China; 7 Jiangsu Collaborative Innovation Center of Chinese Medicinal Resources Industrialization, Nanjing University of Chinese Medicine, Nanjing, 210023 China

**Keywords:** Postoperative wound, Traditional Chinese medicine, Single-cell transcriptomics, Cell interaction

## Abstract

**Background:**

Anal fistula is one of the most common and frequently occurring diseases in the anorectal department. Calvatia lilacina spore (CLS) has been applied for wound treatment with a long history as a traditional Chinese medicine (TCM). However, the mechanism of CLS to treat postoperative wound of anal fistula remains unclear. The present study aims to investigate the efficacy and mechanism of CLS in promoting anal fistula wound healing from the perspective of regulating the interaction between macrophages and fibroblasts.

**Methods:**

Twenty patients who received anal surgery were recruited in Nanjing Hospital of Chinese Medicine Affiliated to Nanjing University of Chinese Medicine. We presented a single-cell atlas of granulation tissue, comparing samples with and without CLS treatment, utilizing single-cell RNA sequencing. The pharmacological effects and mechanism of CLS on anal fistula wound were assessed using elisa, Immunohistochemistry (IHC) staining, western blot, Immunofluorescence (IF) staining, flow cytometry assays and cell co-culture.

**Results:**

The CLS had a uniform particle size and contained components mainly including proteins, steroids, polysaccharides and polyphenols. CLS reduced the expression levels of Tumor Necrosis Factor-alpha (TNF-α) and increased the expression levels of Vascular Endothelial Growth Factor (VEGF) and Collagen Type I Alpha 1 (COL1A1) in the granulation tissue. The single-cell sequencing revealed that the expression level of interleukin 6 (IL-6) and C-X-C Motif Chemokine Ligand 8 (CXCL-8) was increased in the IL-6^+^ macrophages that promoted the expression of Wiskott-Aldrich syndrome protein family member 3 (WASF3) in fibroblasts and further recruited Actin-Related Protein 2 (ACTR2), Actin-Related Protein 3 (ACTR3). Finally, CLS enhanced intercellular communication between macrophages and fibroblasts by activating the Janus Kinase 2 (JAK2)/Signal Transducer and Activator of Transcription 3 (STAT3) signaling pathway, thereby promoting mouse skin fibroblasts (MSF) migration ability.

**Conclusion:**

Our study objectively demonstrated the pharmacological effects of CLS in promoting the wound healing of anal fistula and investigated its mechanisms in terms of regulating the immune inflammatory process of macrophages increases signal communication with fibroblasts while promoting fibroblast transformation.

**Graphical Abstract:**

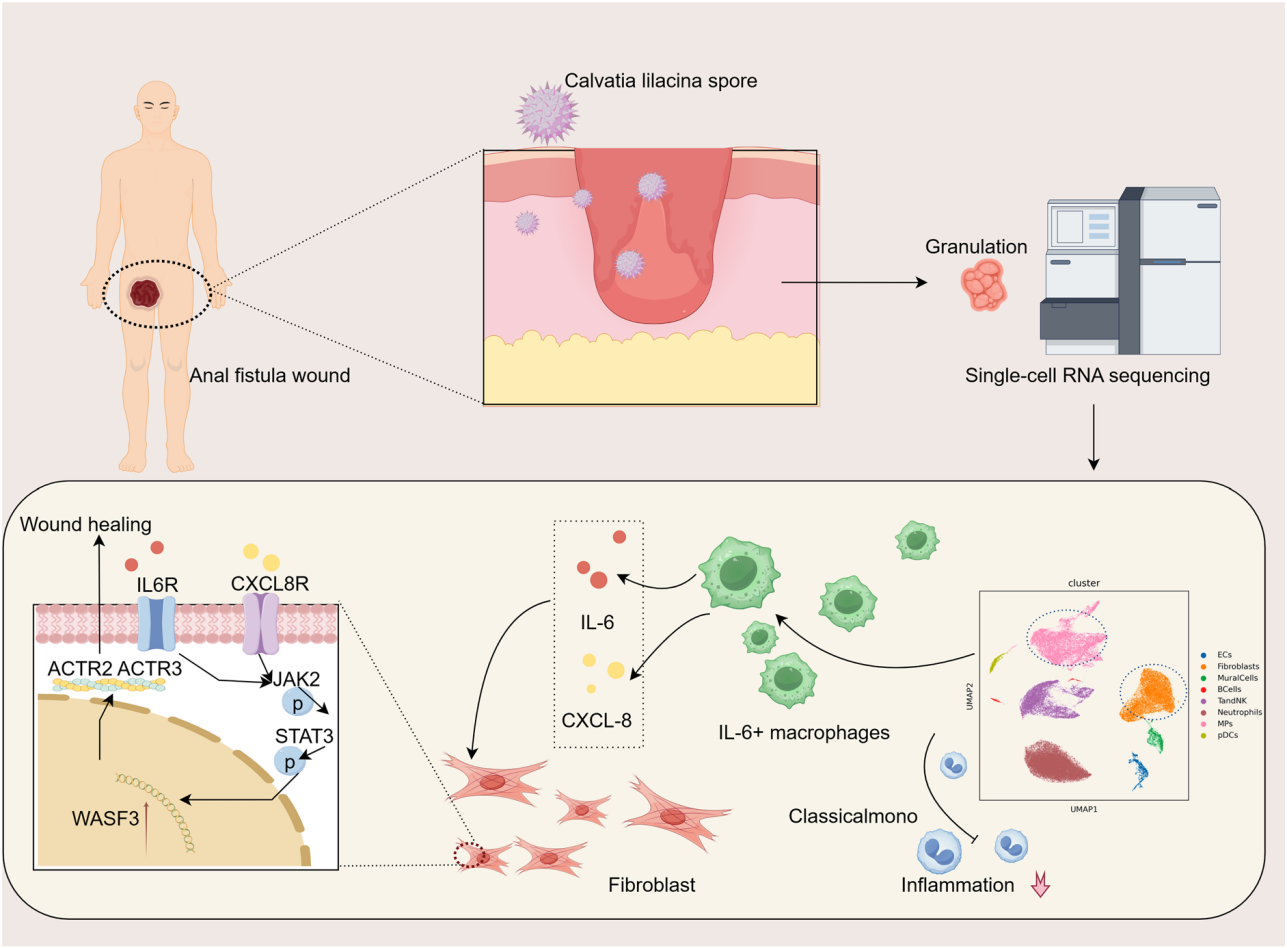

**Supplementary Information:**

The online version contains supplementary material available at 10.1186/s13020-025-01293-w.

## Introduction

Anal fistula is one of the most common and frequently occurring diseases in the anorectal department. The pathogenesis is still unclear, and it has very few well-identified influence factors [1]. Nowadays, the hypotheses such as immune abnormalities [2], an obstruction of anal crypt [3], and bacterial translocation have been proposed [4, 5]. There are also studies indicating a significant correlation between the anal fistula and diet, and daily routine [6]. In clinical settings, patients often experience symptoms such as anal pus discharge, fistula defecation, and pain [7]. The commonly used treatment for anal fistula is surgical removal of the fistula, resulting in the formation of a wound. However, due to the special location of the affected area, the wound is chronic, and there is a strong risk of infection [8], as well as a high recurrence rate after anal fistula surgery [9]. It will be a heavy burden on the patient individual and the whole medical system. Consequently, promoting wound healing plays an essential role in the treatment of anal fistula [10].

The regulation of macrophage and fibroblast functions is the core strategy for wound healing. Macrophages in the early stage engulf pathogens and necrotic tissues, regulate inflammatory responses, and secrete related factors that not only promote angiogenesis, but also activate fibroblast proliferation [11]. During the proliferation phase, they migrate extensively to the wound, synthesize extracellular matrix such as collagen, and differentiate into myofibroblasts to mediate wound contraction, ultimately promoting tissue remodeling and barrier function recovery together [12]. Targeting the signaling pathways of both (such as TGF-β/Smad, Wnt/β-catenin) or intercellular interactions can optimize the repair process, reduce complications, and provide a key entry point for the treatment of wounds [13].

Puffballs are a class of mushrooms that are found worldwide. Some species of puffball are edible when they are fresh, and other species, such as *Calvatia lilacina,* have been used as traditional medicine worldwide. Calvatia lilacina spores (CLS) used as traditional Chinese medicine (TCM) have been proven to be used for the treatment of hemostasis, throat pain and cough [14]. Historical literature used its powder to treat various wounds, and it was mostly used as wound dressings, because dry, mature spores have a positive effect on wound healing [15, 16]. And it had been confirmed that puffballs promoted the healing of diabetes ulcers by balancing oxidative stress, accelerating angiogenesis and regulating wound microbiota [17, 18]. In addition, the CLS extracts had also been shown to inhibit cancer cell proliferation [19]. The CLS has spiky and hollow structure which gives it strong adsorption ability [20]. Therefore, CLS has great potential in the treatment of anal fistula wounds due to its diverse pharmacological effects and unique structures.

The current study sought to evaluate the molecular mechanism of CLS on anal fistula wound. In addition, the study has constructed single-cell transcription maps of granulation tissue from control and treatment groups. Single-cell RNA sequencing analysis enables the identification of cell-type-specific changes of gene expression and high-throughput quantification with individual cells between two groups [21]. Notably, we employed a novel approach to unravel the intercellular heterogeneity of anal fistula granulation tissue and analyzed cell–cell communication using CellChat [22], exploring the key mechanisms by which the complex components and special structure of the TCM CLS promote wound healing.

## Methods and materials

### Reagents and antibodies

TNF-α, COL1A1, VEGF Elisa Kit (EK182, EK183, EK1C01, Multi Sciences, China); WASF3 Antibody (67620-1-lg, Proteintech, China); α-SMA (19245s, CST, USA); CD14 Antibody (ab183322, abcam, Britain); Cluster of Differentiation 11b (CD11b) Antibody (ab133357, abcam, Britain); IL-6 flow Antibody (562050, BD Pharmingen, USA); JAK2 (WL02188, Wanleibio, China); p-JAK2 (WL02997, Wanleibio, China); STAT3 (WL03207, Wanleibio, China); p-STAT3 (WL06214, Wanleibio, China);

### Preparation of CLS, composition analysis and characterization

**Fig. 1:**
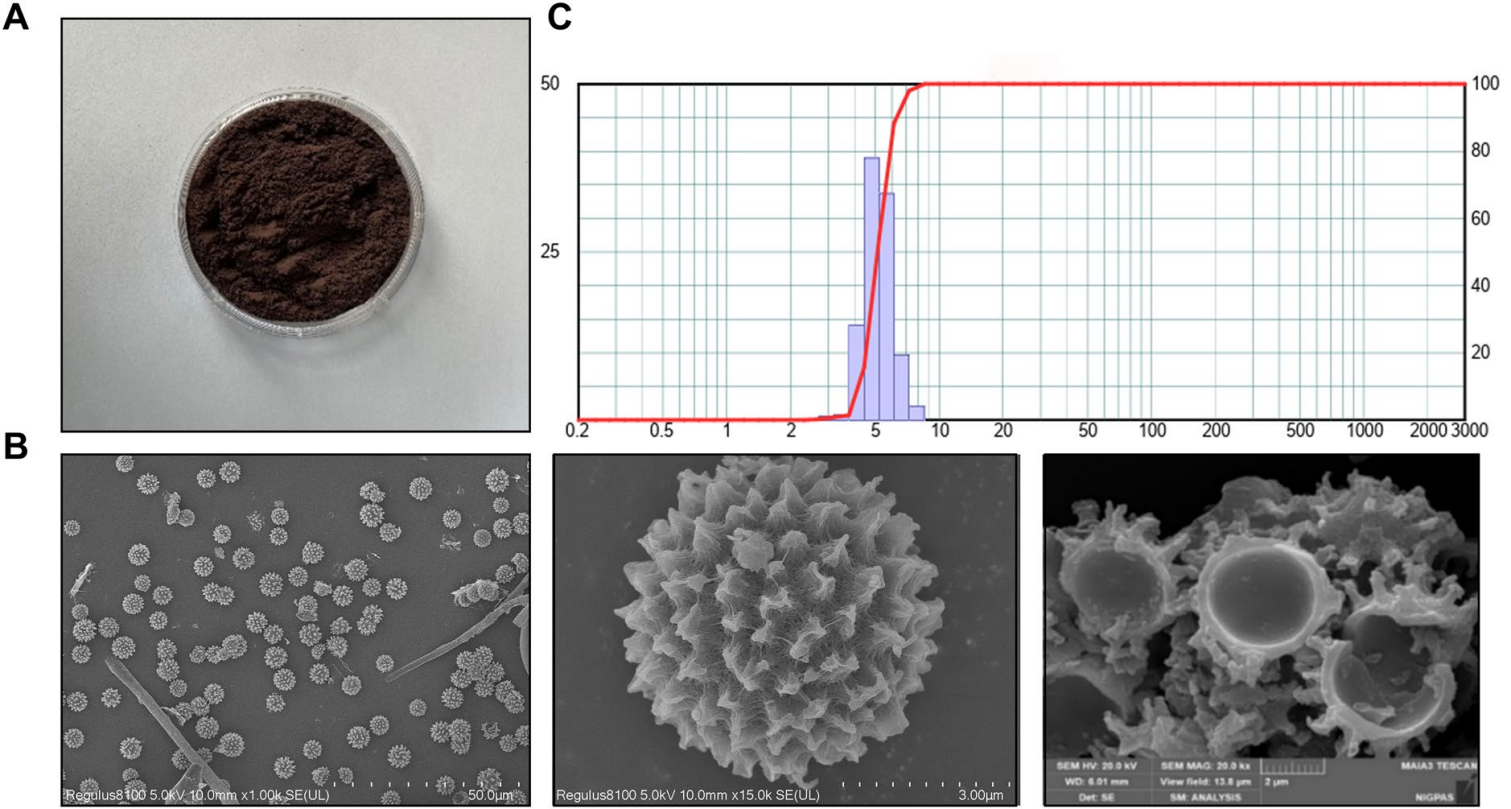
Structural characterization of Calvatia lilacina. **A** CLS powder; **B**, **C** Scanning Electron Microscope (SEM) and particle size analysis of CLS powder."

Calvatia lilacina (Mont.et Berk.) Lloyd was purchased from Anguo TCM Market (Hebei) and authenticated by (blind for peer review) based on the Pharmacopoeia of the People’s Republic of China (Part I, 2020 version). After removing the shell and impurities, the obtained spore powder was sieved through a filter screen and then subjected to radiation sterilization (10 g/bag, ^60^Co-γ 8 kGy, 6 h) for subsequent experiments. Due to its complex composition, we determined the composition of CLS via UV, GC, HPLC and UPLC-MS. In addition, we conducted the CLS morphology related indices and structural characterization (Fig. 1).

### Sample collection

The study enrolled a total of twenty patients who received anal surgery in Nanjing Hospital of Chinese Medicine Affiliated to Nanjing University of Chinese Medicine, and randomly divided into the control group and the treatment group. The control group, patients on the second day after surgery were routinely disinfected with 0.5% iodine cotton balls on the wound, and sterilized vaseline oil gauze was applied to the wound, which was covered and fixed with sterile dry gauze outside. On the basis of the control group, the treatment group evenly applied 0.04 g/cm^2^ of CLS onto sterilized vaseline oil gauze and covered the wound. The dressing was changed twice a day, and collected granulation tissue at different time. The protocol was approved by the Ethics Committee of the hospital (KY2023302). All enrolled participants provided written informed consent to participate in the study under the principles of Declaration Helsinki. In addition, written informed consents were obtained from all the participants before study procedures began, and participants were free to withdraw from the study at any stage.

### Enzyme-linked immunosorbent assays

According to the instructions of the ELISA kit, added 100 μL of detection antibody to the standard and sample wells, except for the blank well, incubated at 37 °C for 1 h. Then, 250–300 μL of washing solution was added to each well for 1 min, discarded the liquid. Subsequently, each well added color reagent and incubated in the dark for 15 min. Afterwards, added 50 µL of stop solution to the plate. Finally, the OD value was determined by the enzyme marker at 450 nm.

### IHC staining of granulation tissues

Obtaining granulation tissue slides consistent with the above method for subsequent detection of CD14 and CD11b. After dewaxed and dehydrated the tissue slides, performed antigen repair at high temperatures, they were blocked with serum, then incubated overnight at 4 ℃ with primary antibody. Secondary antibodies corresponding to the species of the primary antibodies were then incubated. Stained the cell nucleus with hematoxylin, slides dehydrated, sealed and then examined with a microscope. The expression of specific proteins be analyzed by detecting the mean gray value of immunohistochemistry photographs.

### Tissue dissociation and preparation for single-cell RNA sequencing

The fresh tissues were stored in the sCelLiveTM Tissue Preservation Solution (Singleron) on ice after the surgery within 30 min. The specimens were washed with Hanks Balanced Salt Solution (HBSS) for three times, minced into small pieces, and then digested with 3 mL sCelLiveTM Tissue Dissociation Solution (Singleron) by Singleron PythoN™ Tissue Dissociation System at 37 °C for 15 min. The cell suspension was collected and filtered through a 40-micron sterile strainer. Afterwards, the GEXSCOPE^®^ red blood cell lysis buffer (RCLB, Singleron) was added, and the mixture [Cell: RCLB = 1:2 (volume ratio)] was incubated at room temperature for 5–8 min to remove red blood cells. The mixture was then centrifuged at 300 × g 4 ℃ for 5 min to remove supernatant and suspended softly with PBS.

### RT & amplification & library construction

Single-cell suspensions (2 × 10^5^ cells/mL) with PBS (HyClone) were loaded onto microwell chip using the Singleron Matrix^®^ Single Cell Processing System. Barcoding Beads are subsequently collected from the microwell chip, followed by reverse transcription of the mRNA captured by the Barcoding Beads and to obtain cDNA, and PCR amplification. The amplified cDNA was then fragmented and ligated with sequencing adapters. The scRNA-seq libraries were constructed according to the protocol of the GEXSCOPE^®^ Single Cell RNA Library Kits (Singleron) (Ref). Individual libraries were diluted to 4 nM, pooled, and sequenced on Illumina novaseq 6000 with 150 bp paired end reads.

### Primary analysis of raw read data

Raw reads were processed to generate gene expression profiles using CeleScope V3.0.1 (Singleron Biotechnologies) with default parameters. Briefly, Barcodes and UMIs were extracted from R1 reads and corrected. Adapter sequences and poly A tails were trimmed from R2 reads and the trimmed R2 reads were aligned against the GRCh38 (hg38) transcriptome using STAR (v2.6.1b). Uniquely mapped reads were then assigned to genes with FeatureCounts(v2.0.1). Successfully Assigned Reads with the same cell barcode, UMI and gene were grouped together to generate the gene expression matrix for further analysis.

### Quality control, dimension-reduction and clustering

Scanpy v1.8.1 was used for quality control, dimensionality reduction and clustering under Python 3.7. For each sample dataset, we filtered expression matrix by the following criteria: (1) cells with gene count less than 200 or with top 2% gene count were excluded; (2) cells with top 2% UMI count were excluded; (3) cells with mitochondrial content > 10% were excluded; (4) genes expressed in less than 5 cells were excluded. After filtering, 81068 cells were retained for the downstream analyses, with on average 1004 genes and 2183 UMIs per cell. The raw count matrix was normalized by total counts per cell and logarithmically transformed into normalized data matrix. Top 2000 variable genes were selected by setting flavor = ‘seurat’. Cells were separated into 22 clusters by using Louvain algorithm and setting resolution parameter at 1.2. Cell clusters were visualized by using Uniform Manifold Approximation and Projection (UMAP). Batch effect between samples was removed by Harmony v1.0.

### Cell–cell interaction analysis

CellChat (version 0.0.2) was used to analyze the intercellular communication networks from scRNA-seq data. A CellChat object was created using the R package process. Cell information was added into the meta slot of the object. The ligand-receptor interaction database was set, and the matching receptor inference calculation was performed.

### Pseudotime trajectory analysis: monocle2

Cell differentiation trajectory of Fibroblasts subtypes was reconstructed with the Monocle2 v 2.10.0 (ref). For constructing the trajectory, top 2000 highly variable genes were selected by Seurat(v3.1.2) FindVairableFeatures() and dimension-reduction was performed by Discriminative Dimensionality Reduction Tree (DDRTree)(). The trajectory was visualized by plot_cell_trajectory() function in Monocle2.

### UCell gene set scoring

Gene set scoring was performed using the R package UCell v 1.1.0. UCell scores are based on the Mann–Whitney U statistic by ranking query genes’ in order of their expression levels in individual cells. Because UCell is a rank-based scoring method, it is suitable to be used in large datasets containing multiple samples and batches.

### Culture and intervention of Tohoku Hospital Pediatrics-1 (THP-1)

THP-1 cells were cultured in RPMI-1640 medium (Gibco) supplemented with 10% fetal bovine serum (FBS), 100 U/mL penicillin, and 100 μg/mL streptomycin in a 5% CO₂ incubator at 37 °C. Cell selection during logarithmic growth phase, added phorbol 12-myristate 13-acetate (PMA, 500 nM) and induced for 24 h in a 12 well plate with a concentration of 10^6^ cells/ml. Then the control group was given single culture, while the CLS group was incubated with particles at different dosages 0.04, 0.08 particles/μm^2^, the SiO_2_ group was 0.08 particles/μm^2^ for 24 h. Then cells and culture supernatant were collected for subsequent experiments.

### Culture of MSF with conditioned medium and transewell migration assay

The supernatant of the four groups of macrophages were mixed with culture medium, to form conditioned medium. After culturing 24 h, proteins were collected for subsequent detection. Meanwhile, 500 μL of the conditioned medium was added to the lower chamber, and 100 μL of MSF suspension (2 × 10^5^ cells/ml) was added to the upper Transwell chamber. After 24 h of incubation, the cells were fixed with methanol for 15 min and stained with 1% crystal violet for 15 min. After washing with PBS, wiped off the cells in the small chamber with a cotton swab, and finally took photos and performed quantitative analysis.

### Flow cytometry

Adherent cells were incubated with trypsin EDTA at 37 °C for 1 min and gently dislodged by pipetting for cell harvest, washed the cells twice with PBS buffer. Then 1 μl of the BD Horizon Fixable Viability Stain 660 Stock Solution was added for 10 min. Nonspecific binding of cell surface antigens was blocked by incubation of the cells with Fc Block. The cells were fixed, permeabilized then subsequently stained with 0.06 µg of PE-rat anti-mouse IL-6 antibody at 4 °C for 30 min. Finally, the cells were centrifuged, the supernatant was discarded, washed three times with PBS, and resuspended in 250 μl PBS for fluorescence intensity detection.

### IF staining of granulation tissues

Granulation tissue slides were subjected to antigen retrieval and then blocked with bovine serum albumin (BSA). Incubating overnight with WASF3 primary antibody (proteintech, 67620-1-Ig, 1:500), followed by secondary antibody incubation and DAPI staining. Then added anti-fluorescence quencher and sealed the slide for fluorescence microscopic examination. The gray value of each pixel represents the fluorescence intensity of the point, and the mean gray value of a certain fluorescence channel on the immunofluorescence photograph can be quantified using ImageJ.

### Western blot

The conditioned medium formed by macrophage culture was added to MSF and cultured for 24 h. Cell proteins were extracted with RIPA lysis buffer and separated by sodium dodecyl sulfate polyacrylamide gel electrophoresis. After electrophoresis, the proteins were transferred onto the PVDF membrane, blocked it with a blocking solution for 1 h, and then washed it with TBST. The membrane was incubated with the primary antibody overnight at 4 °C. The next day, the membrane was washed with TBST and incubated with the corresponding secondary antibody at room temperature for 1 h. Protein bands were visualized using enhanced chemiluminescence (ECL) reagent, and the grayscale values were calculated using ImageJ.

### Statistical analysis

The statistical analysis was made by SPSS 22.0 software. Descriptive statistics were reported as mean ± SD and P-value of less than 0.05 was considered significant.

## Results

### Component analysis and structural characterization

In this study, the results showed that CLS contained total protein, total steroids, total polysaccharides and total polyphenols in CLS (Table 1, supplementary results S1-4). For fatty acid analysis, GC-2010 plus was used. The saturated fatty acid content was analyzed as 0.2007% (C16:0 methyl palm 0.1270%, C18:0 stearic acid methyl ester 0.0616%, C24:0 methyl tetracosanoate 0.0121%). Monounsaturated fatty acids and polyunsaturated fatty acids were analyzed as 0.3050% and 0.0815% (C18:1n-9c methyl 9-oleate acid methyl ester, C18:2n-6c linoleic acid methyl ester) (Supplementary Table 1, S7). Cysteine (0.1465%) and valine (0.0803%) were determined as major components of amino acids in the CLS (Supplementary Table 2). UPLC-MS and HPLC detected the representative monomeric components ergosterol (0.142%) and ergosterone (0.023%) in CLS (Supplementary results S5, S6). The spore powder has a uniform particle size and is spiky (Fig. 1). The micromeritic characteristics of CLS were also observed (Supplementary Table 3).

**Table 1:** Summary of the contents of total steroids, total polyphenols, total polysaccharides, and total protein in the CLS (mean ± SD, n = 3)

Contents	Total protein (contain amino acids, mg/g)	Total steroids (mg/g)	Total polysaccharides (mg/g)	Total polyphenols (mg/g)
Calvatia lilacina	26.32 ± 0.71	15.15 ± 0.45	11.03 ± 0.54	8.53 ± 0.32

### CLS promoted wound healing in anal fistula by reducing TNF-α-driven inflammation and promoting Collagen I Synthesis, angiogenesis

**Fig. 2:**
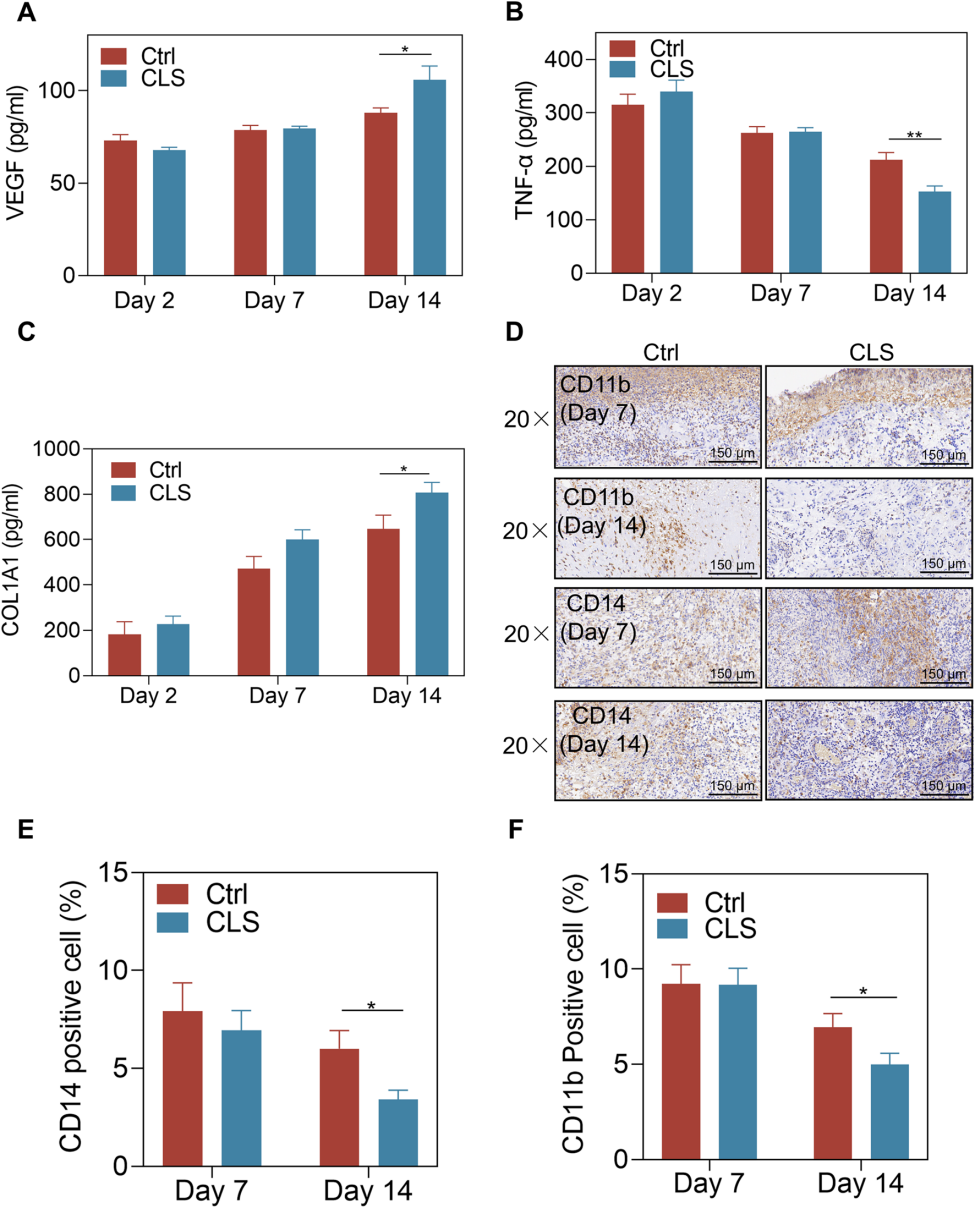
The impact of CLS on inflammation, angiogenesis, and collagen at different time points. **A**–**C** ELISA detection of TNF-α, VEGF, and Collagen I expression levels in granulation tissue; **D** Immunohistochemical staining of CD14 and CD11b; **E**, **F** Immunohistochemical quantitative analysis. *P < 0.05, **P < 0.01, ***P < 0.001, ****P < 0.0001, and ns: not significant (*P* > 0.05) vs.the control group; n = 10

To verify the role of CLS in collagen generation, angiogenesis, and inflammation, we detected the expression levels of TNF-α, VEGF, and Collagen I in granulation tissue by ELISA. As shown in the results (Fig. 2A–C), CLS significantly inhibited the levels of inflammatory cytokine TNF-α with the most significant effect observed on day 14. Meanwhile, CLS also promoted the levels of type I collagen, a key component for collagen generation, and had a slight effect on the expression of the vascular factor VEGF. We further performed immunohistochemical staining on granulation tissue on days 7 and 14 to observe the status of inflammation-related cells (Fig. 2D). Consistent with the ELISA results, there was a significant decrease in the positive expression rates of CD14 and CD11b on day 14. As shown by histological results, strong positive expression was observed on days 7 and 14, indicating that immune and inflammation related cells are the main functional groups in the early stage of anal fistula wound healing (Fig. 2E, F).

### Single-cell atlas of anal fistula granulation tissue revealed macrophages were the main cell population of MPs

**Fig. 3:**
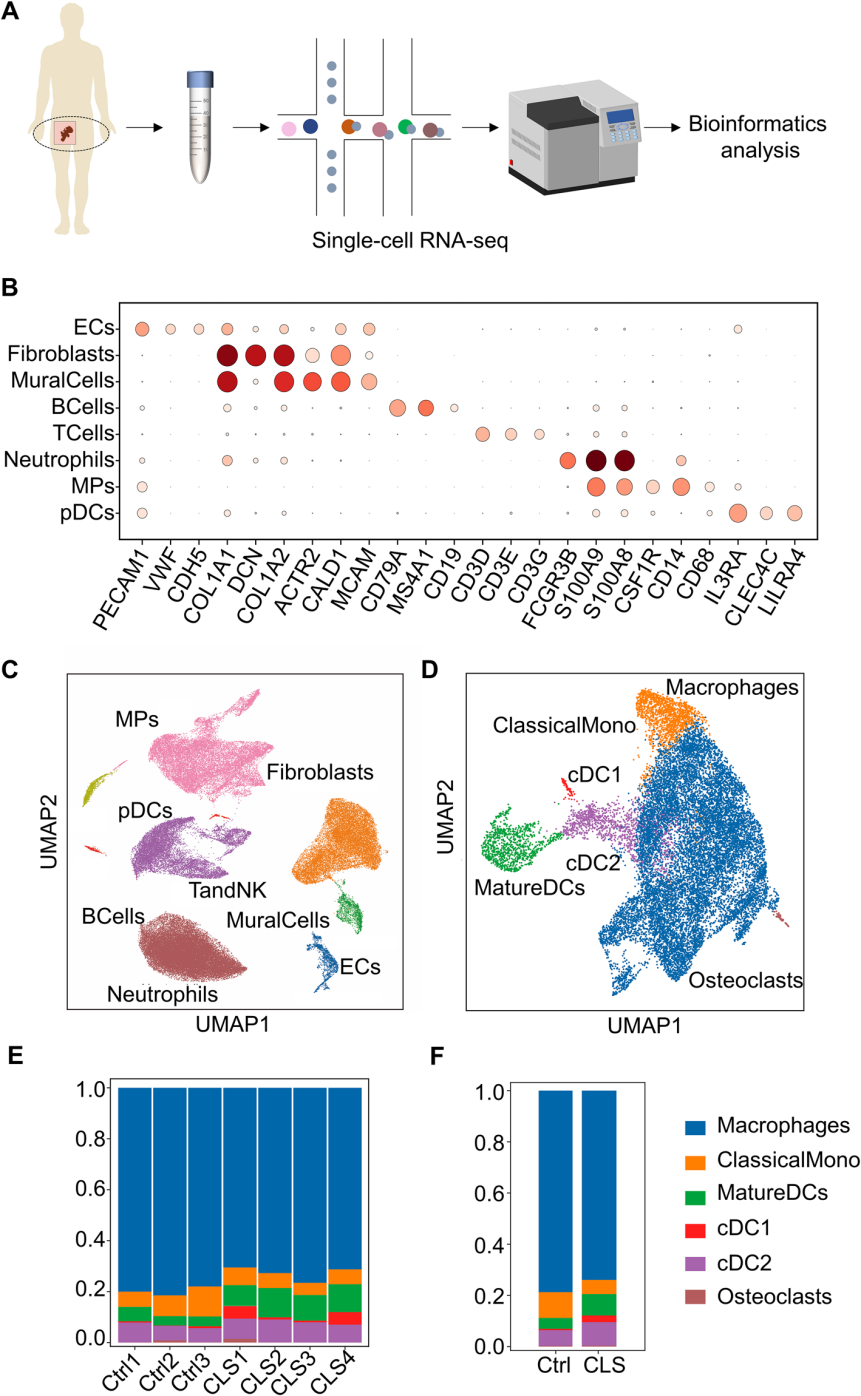
Single-cell RNA-seq analysis of granulation tissue samples reveals the cellular landscape of anal fistula wound healing. **A** Overview of the scRNA-seq samples from granulation tissue; **B**, **C** Uniform Manifold Approximation and Projection (UMAP) plot for dimension reduction of all cells colored by their cell type/identity and example marker genes are highlighted, dark represents high expression; light represents low expression; **D** UMAP plot of different MPs colored by their cell type in two groups; **E**, **F** Bar charts of the proportion of different MPs clusters in two groups

To obtain a better understanding of the effect of CLS on the makeup of cell types and transcriptional patterns in granulation tissue, we created single-cell atlases for the granulation tissue. GEXSCOPE^®^ was used to convert single-cell suspensions of the scRNA-seq samples to barcoded libraries. A total of 81068 viable cells were obtained after quality control for subsequent analyses. To identify each cell type, we used the Seurat v2.3.4 packages to perform clustering on the sequencing data, then annotated each cell type based on the expression levels of canonical cell-type-specific markers. In general, we identified 22 cell clusters in granulation tissue that could be classified into eight major cell types: ECs (identified by PECAM1, VMF, CDH5), Fibroblasts (identified by COL1A1, DCN, COL1A2), MuralCells (identified by ACTA2, CALD1, MCAM), BCells (CD79A, MS4A1, CD19), TCells (CD3D, CD3E, CD3G), Neutrophils (FCGR3B, S100A9, S100A8), MPs (CSF1R, CD14, CD68), pDCs (IL3RA, CLEC4C, LILRA4) (Fig. 3A–C). In summary, we developed a tissue characterization of the cellular diversity of the granulation tissue and established a comprehensive framework.

Single-cell results showed a significant reduction in the number of macrophages and monocytes in the treatment group, indicating that CLS had a certain anti-inflammatory effect in the early stage (Fig. 3D–F). Therefore, we focused on macrophages as the main cell population for further dimensionality reduction analysis.

### CLS induced an IL-6^+^ macrophage subset with coordinated expression of IL-6 and CXCL-8 during wound healing

**Fig. 4:**
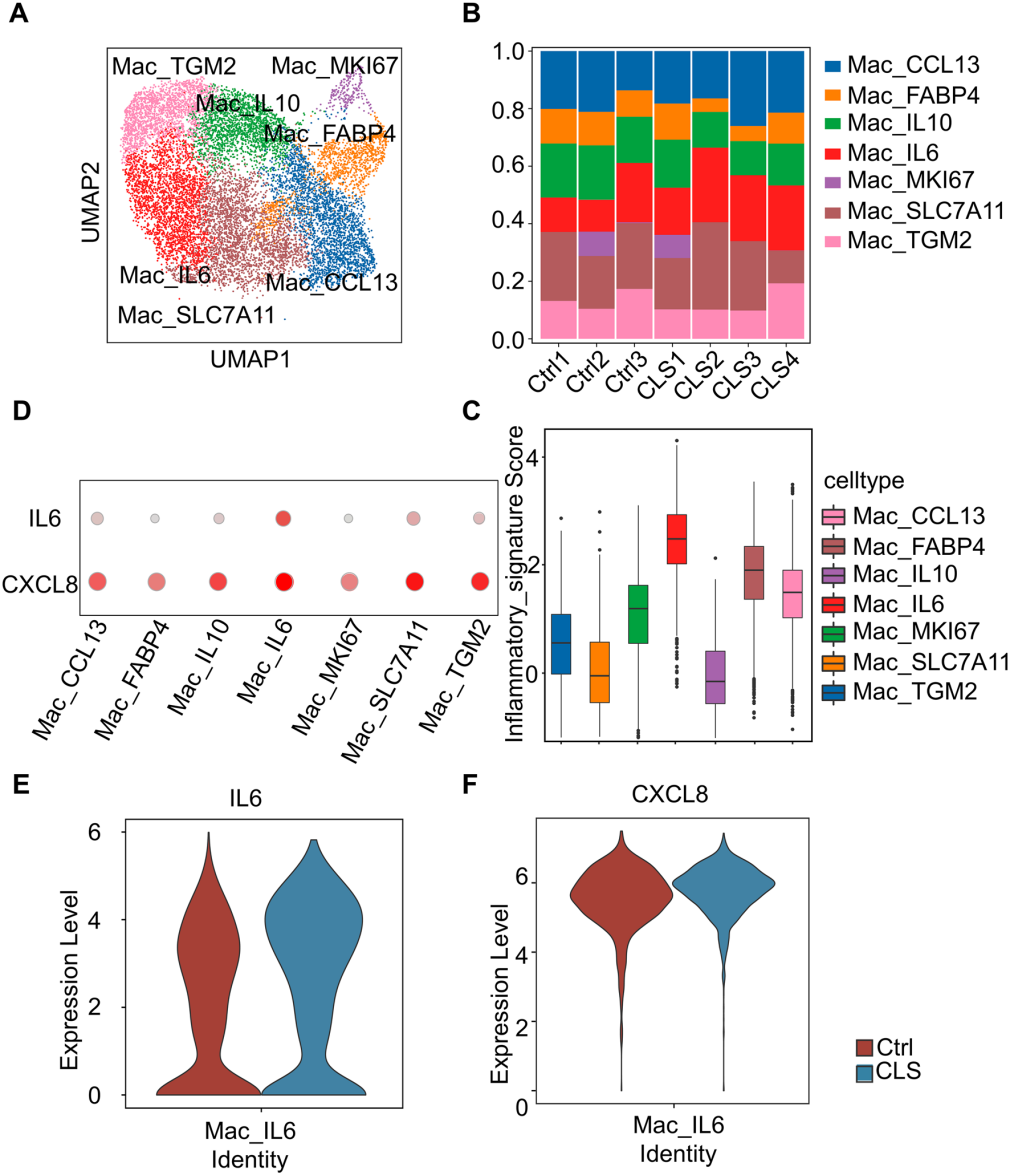
Clustering and functional annotation of macrophage. **A** UMAP plot of different macrophages colored by their cell type; **B** Bar charts of the proportion of different macrophages clusters in two groups; **C** Boxplots of inflammatory by macrophages subtypes; **D** The expression of IL-6, CXCL-8 in macrophages subtypes; **E**, **F** Violin plot of IL-6, CXCL-8 distribution in IL-6^+^ macrophages across 2 groups

A total of 13023 viable macrophages were obtained to recluster on the sequencing data, and we identified 7 cell clusters in macrophages. The results showed that there was a certain increase in the number of IL-6^+^ macrophages, meanwhile IL-6^+^ macrophages regulate inflammation function significantly stronger than other subgroups (Fig. 4A–C). Research report suggested that the coexistence of IL-6 and CXCL-8 in macrophages could activate the WASF3 [23]. Therefore, we further compared the expression of IL-6 and CXCL-8 in the macrophage. The results showed that the IL-6 and CXCL-8 had co-high expression only in IL-6^+^ macrophages (Fig. 4D). This also suggested that IL-6^+^ macrophages may be a special cell population after CLS intervention. The results further showed that the expression of IL-6 and CXCL-8 was significantly increased in the treatment group (Fig. 4E, F).

### CLS enhanced macrophages and fibroblasts communication through IL-6R and CXCL-8R1 receptors

**Fig. 5:**
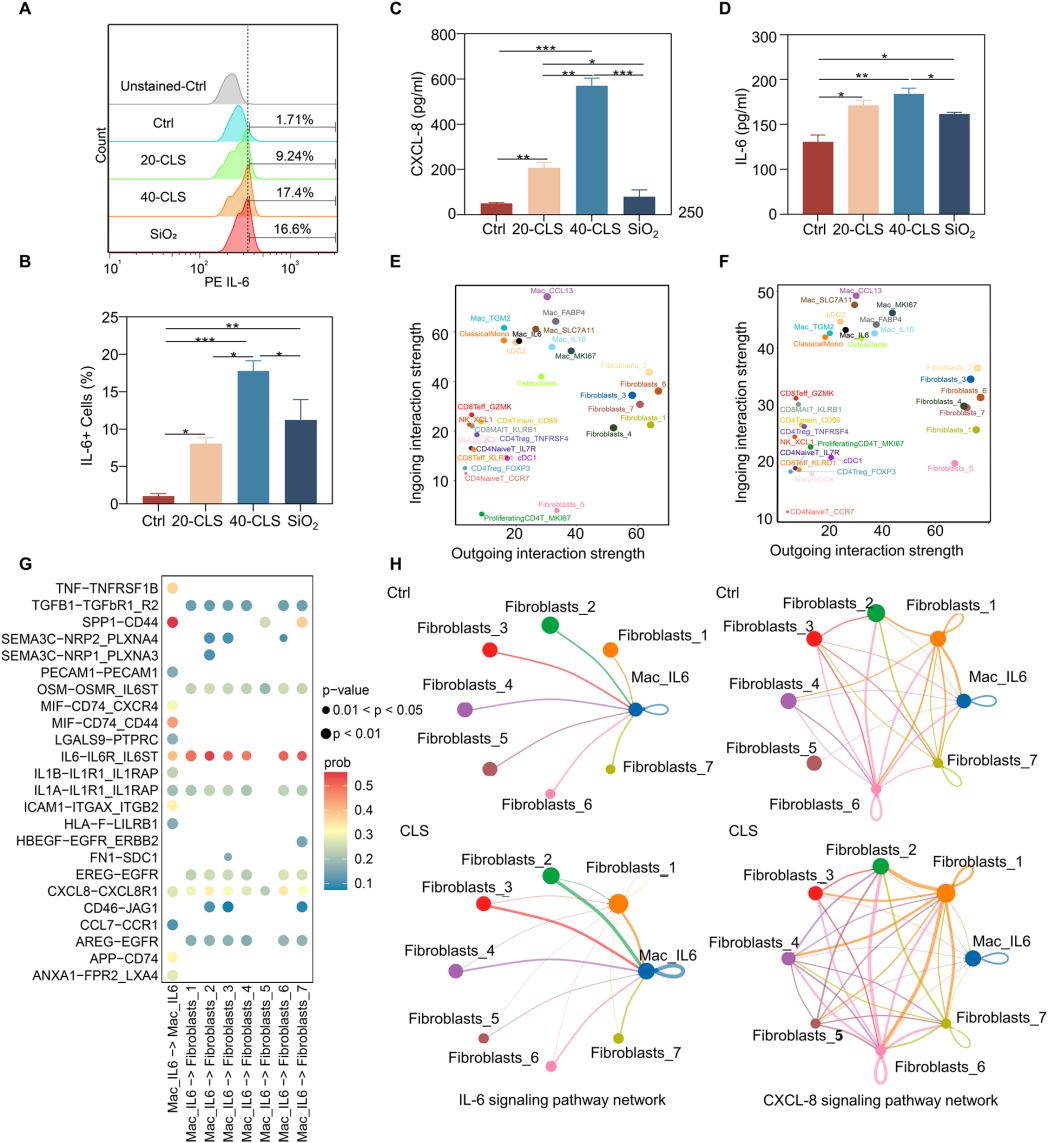
Flow cytometry and SEM detection of adhesion and effect of CLS on macrophages. **A** Flow cytometry was used to determine the ratio of IL-6 positive cells in macrophage after extraction. **B** Quantitative analysis the rate of IL-6 positive macrophage. **C**, **D** Elisa detection of IL-6 and CXCL-8 in cell supernatant. **E**, **F** Intensity of intercellular communication between control group and treatment group. **G** Analysis of IL-6^+^macrophage and fibroblast receptor-ligand binding. **H** Analysis of cell population interaction intensity of IL-6, CXCL-8 signaling pathway. *P < 0.05, **P < 0.01, ***P < 0.001, and ns: not significant (*P* > 0.05) vs.the control group; n = 3

CLS adhered to macrophages and spike SiO_2_ also had the effect of adhering to cells. The flow cytometry results showed that CLS increased the number of IL-6^+^ macrophages, and the SiO_2_ group also had a certain effect (Fig. 5A, B). This result reminded us that the spike structure of CLS could stimulate immune activity which is consistent with previous reports [24]. The elisa results showed that CLS significantly increased the expression levels of IL-6 and CXCL-8 (Fig. 5C, D). In addition, studies have shown that ergosterol can activate the signal expression of AP-1 protein and AP-1 is required for CXCL-8 activation [25]. Meanwhile, we found CLS enhanced the strength of cell interaction between macrophages and fibroblasts (Fig. 5E, F). The gene sequence of mouse C-X-C motif chemokine ligand 1 (CXCL-1) had high homology with human CXCL-8 at the amino acid level. The elisa results showed that CLS significantly increased the expression levels of IL-6 and CXCL-1 (supplementary results S8). IL-6 and CXCL8 activated downstream signaling pathways by binding to receptors, the results showed that there were multiple receptors for IL-6^+^macrophages in fibroblasts, among which IL-6R_IL-6ST and CXCL-8R1 were the main functional receptors. CLS significantly enhanced the inter-population communication intensity of IL-6 and CXCL-8 signaling pathways (Fig. 5G, H).

### CLS promoted macrophages to co-upregulate IL-6 and CXCL-8, thereby accelerating downstream WASF3-mediated wound closure

**Fig. 6:**
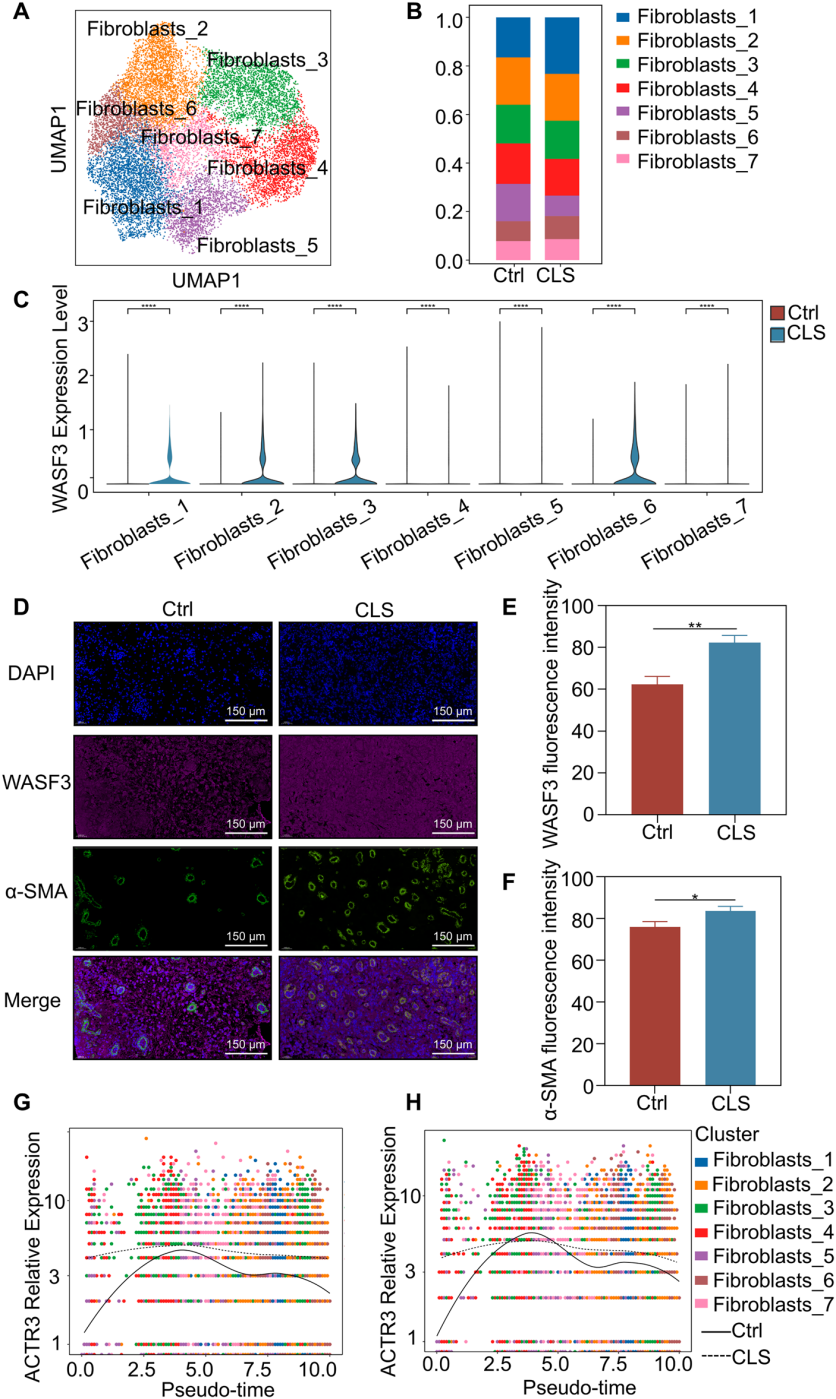
Clustering annotation of fibroblast and changes of the key regulatory factors of wound closure. **A** UMAP plot of different fibroblast colored by their cell type; **B** Bar charts of the proportion of different fibroblast clusters in two groups; **C** Violin plot of WASF3 distribution across the control and treatment groups; **D** Immunofluorescence staining of WASF3 and α-SMA; **E, F** Immunofluorescence quantitative analysis. **G**, **H** Cluster-defined fibroblast related gene expression of different subclusters on pseudo timeline; *P < 0.05, **P < 0.01, and ns: not significant (*P* > 0.05) vs.the control group; n = 10

During the process of wound healing, fibroblasts are the main cell group to regulate wound closure [26]. We subset fibroblasts for reintegrating and subclustering. Altogether, 15679 cells contributed to 7 clusters (Fig. 6A, B). We found that the expression of ACTR2, ACTR3 and WASF3 in the treatment group was significantly higher than the control group (Fig. 6C, supplementary results S9 D,E). Our further immunofluorescence results showed that after CLS intervention, the expression levels of α-SMA and WASF3 increased in granulation tissue, consistent with single-cell detection data (Fig. 6D-F). The gene expression pattern across pseudotime showed that the expression curves of ACTR2 and ACTR3 were relatively smoother in the treatment group than in the control group. Meanwhile, the overall expression level of ACTR2 in the treatment group was higher than that in the control group (Fig. 6G, H). ACTR2, ACTR3 are components of the Arp2/3 complex, which is involved in regulating various cellular processes.

WASF3 plays a critical role in regulating actin cytoskeleton dynamics, and previous study showed that cell migration through increased the expression of WASF3 to recruit the Arp2/3 complex, increasing the formation of dendritic protrusions and thus driving cell migration [23]. Therefore, we reasonably speculated that CLS enhanced the expression of IL-6 and CXCL-8 in the IL-6^+^ macrophage, thereby increasing the expression level of WASF3 in fibroblasts and accelerating wound closure.

### The conditioned medium derived from macrophage cultures stimulated MSF migration by activating the JAK2/STAT3 signaling pathway

**Fig. 7:**
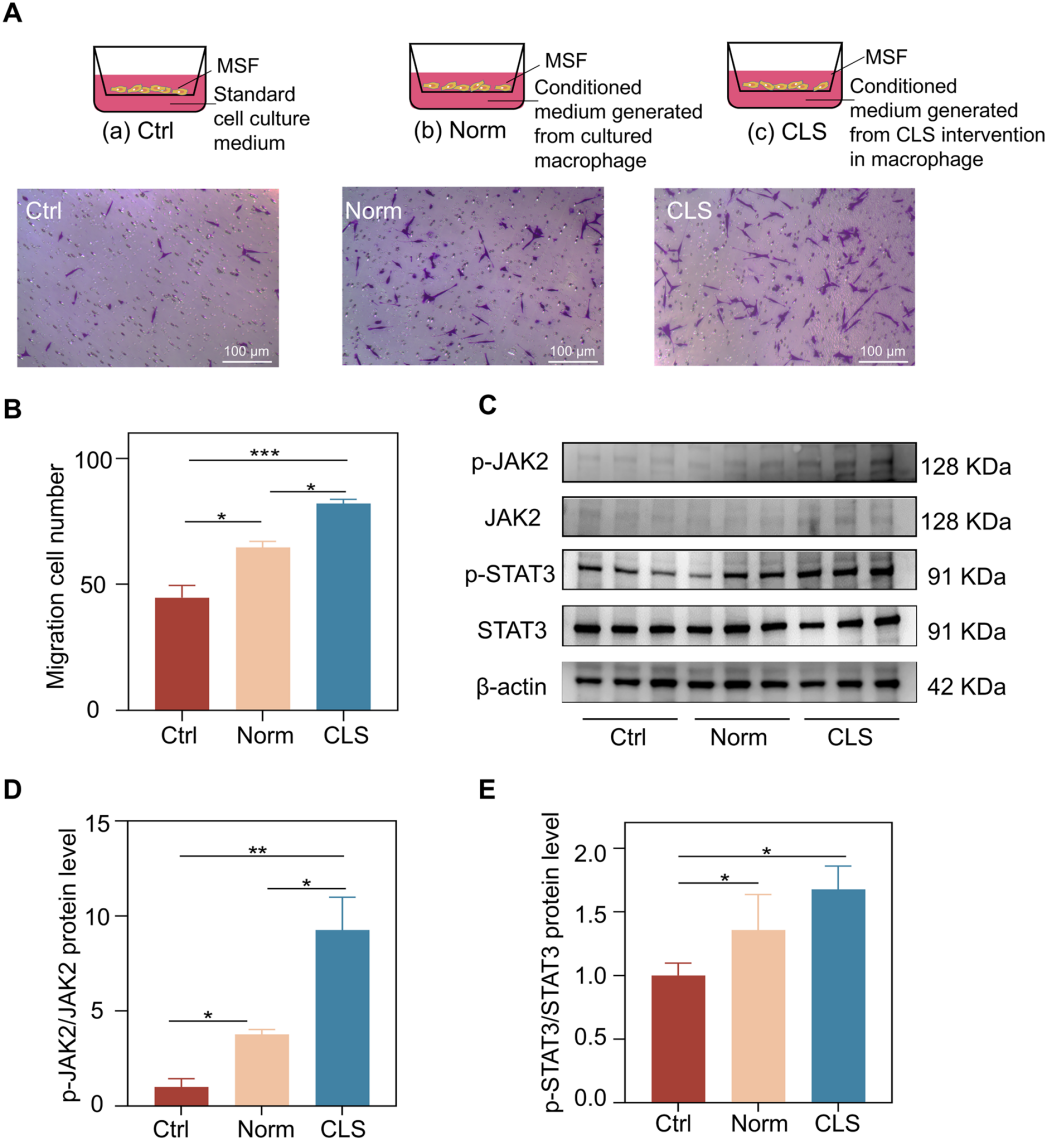
Detection of cell migration ability and related signaling pathways protein expression. **A** Schematic diagram and results of the coculture and grouping of MSF with the conditioned medium for macrophage culture using Transwell constructs. **B** Quantitative analysis of cell migration quantity. **C**–**E** Protein levels of JAK2, p-JAK2, STAT3 and p-STAT3 protein levels in MSF. *P < 0.05, **P < 0.01, ***P < 0.001, ****P < 0.0001, and ns: not significant (*P* > 0.05) vs.the control group; n = 3

The Transwell experiment results showed that the conditioned medium for macrophage culture promoted the migration of MSF (Fig. 7A, B). The JAK2/STAT3 signaling pathway regulates multiple cell functions, such as proliferation, differentiation, migration and immune function. Several studies have confirmed that activated JAK2/STAT3 signaling pathway can promote wound healing [27]. Therefore, we next investigated whether the migration effect of CLS involved the JAK2/STAT3 pathway. To this point, our data indicated that CLS activated the JAK2/STAT3 pathway. As a classic prosurvival signaling axis, the JAK2/STAT3 pathway possesses the capacity to promote cell migration. In addition, the western blot results showed that CLS promoted the phosphorylation of JAK2/STAT3 (Fig. 7C, D), thereby promoting the expression of downstream protein WASF3 to accelerate cell migration.

## Discussion

Due to the complexity of the pathogenesis of anal fistula, there is a huge challenge in promoting wound healing after surgical treatment. In clinical, there are no specific drugs for anal fistula wounds and most of them are only given conventional drugs after debridement to promote healing [28]. Fumigation, rubbing and external washing are common treatment methods, which greatly increases the pain of dressing changes for patients. In addition, the special location of the anal fistula wound often leads to poor treatment outcome [29]. Puffballs can promote the healing of diabetes ulcers through multiple components and targets, which is confirmed by our previous research [17]. In this study, we demonstrated that the CLS offered a safe and well-tolerated treatment option for anal fistula wounds.

Spore powder is an amazing substance in nature that plays a fundamental role in the plants' reproduction. Spore powder is very uniform in size and shape. According to their particular structure, spore powder have essential characteristics such as acid and alkyl resistance, mucosal adhesion, and great stability [30]. Based on this, spore powder is increasingly being used for disease treatment. Spore powder can target the immune system, potentially causing allergies or stimulating inflammatory responses through related mechanisms. Ganoderma lucidum spore powder (GLSP) could regulate CD4^+^CD8^+^T cell ratio and macrophage function, especially the expression of PD-1 in TIME [31]. Inonotus hispidus spore powder (IHS) influenced T cells in ApcMin/^+^ mice by regulating the IL-5, -6, and -10 levels, thus suppressing tumor development [32]. Spore-mimetic metal–organic frameworks (MOFs) had greater cell attachment and faster and more efficient phagocytosis in cells, which resulted in greater expression levels of pro-inflammatory cytokines [33].

Excessive inflammation and impaired fibroblast function are important reasons why wounds are difficult to heal [34]. Inflammatory and immune related cells play an important role as the main functional cell groups in the early stage of wound healing. And CLS increased the expression level of IL-6 and CXCL-8 in the IL-6^+^ macrophage. Previous research had shown that ERK3-mediated CXCL-8 secretion was critical for the chemotaxis of inflammatory cells to the epithelium, locally used recombinant protein accelerated wound healing [35, 36]. And furthermore, the study demonstrated that IL-6 signaling was required for chemotaxis and promoted generation of a new subset of tissue repair macrophages [37, 38]. Importantly, IL-6 and CXCL-8 promoted the migration of MCF10A cells and HER2-positive breast cancer cells [39]. These suggested IL-6 and CXCL-8 could promote migration in the neighboring cell and influence the migratory behavior of the entire wound microenvironment. Palmitate in fatty acids had the ability to promote IL-6 via coordinated acetylation of H3K9/H3K18, p300, and RNA Polymerase II [40]. Steroids are crucial components of anti-inflammatory effects by neutralizing pro-inflammatory cytokine production in human monocytes, and are also found in CLS constituents such as ergosterol and ergosterone [41].

CLS simultaneously regulated the function of IL-6^+^ macrophages that enhanced mutual communication with fibroblasts to promote wound contraction. Wound contraction is a process that occurs during the healing of an open wound. Modified fibroblasts were first observed in the granulation tissue of healing wounds which led to the suggestion that these cells have a role in the production of the contractile force that is involved in this process. The myofibroblast had a role in the synthesis of extracellular matrix (ECM) and in force generation, which resulted in ECM reorganization and wound contraction [42]. Related research showed that polysaccharides effectively promoted the formation of granulation tissue, collagen deposition [43]. Polyphenols inhibited collagenase inhibitory activities, which play a critical role in preventing collagen breakdown [44]. It is instructive to compare the mechanism of CLS with other TCM commonly used for wound healing. For instance, Astragalus membranaceus is primarily known for enhancing systemic immunity and promoting angiogenesis, while Salvia miltiorrhiza exerts its effects mainly through anti-inflammatory activity and improving microcirculation [45, 46]. In contrast, CLS uniquely promotes wound repair through multicellular crosstalk between macrophages and fibroblasts, mediated by the IL-6/CXCL-8-JAK2/STAT3-WASF3 axis. Furthermore, while the synergistic effect of the entire spore is likely crucial, key bioactive components in CLS, such as ergosterol, may play a predominant role in its regulation of inflammatory and pro-migratory effects, as suggested by its established ability to modulate cytokine production and signaling pathways involved in wound healing.

A recent study observed that saliva promoted fibroblast migration by increasing the secretion of IL-6 and CXCL-8 [47]. Consistent with this, our research firstly found that CLS promoted signal exchange between fibroblasts and macrophages, which increased the expression level of IL-6 and CXCL-8 in the IL-6^+^ macrophage, then promoted the expression of WASF3 in fibroblasts via JAK2/STAT3 pathway, thereby promoting wound contraction and accelerating wound healing. Unlike previous studies that focused on wound healing regulated by alterations in a single cell type, our study was the first to reveal that CLS promotes wound contraction by enhancing intercellular communication between IL-6⁺ macrophages and fibroblasts and activating the JAK2/STAT3–WASF3 axis. This multicellular coordination mechanism provides a new perspective on the therapeutic action of natural products in complex wound healing environments.

Our metabolic analysis showed that CLS significantly enhances lysine degradation, glycosaminoglycan biosynthesis-keratin sulfate, D-glutamine and D-glutamate metabolism, and selenocompound metabolism had a significant impact (supplementary results S10 A-C). Lysine degradation was an important metabolic process and research had confirmed that lysine was able to synthesize glutamate [48]. More recent work indicates that fibroblasts are the primary producers of collagen and synthesize proline from glutamate rather than arginine. Glutamate is known to be the core of almost all metabolic pathways required at different stages of inflammatory wounds. We have already demonstrated in our previous research that glutamate promoted the proliferation of human keratinocyte (HaCaT) cells, migration of MSFs and macrophage polarization [18]. The fibroblasts synthesize collagen and Glycosaminoglycans (GAGs) which are components of the ECM. Myofibroblasts produced keratan sulfate which assisted in collagen polymerization [49].

This study had several limitations. The treatment group had the effect of promoting angiogenesis and collagen regeneration on the 14th day. Previous research showed that CXCL-8 stimulated angiogenesis in the skin [36]. The causal relationship and specific mechanism still need to be clarified. Notwithstanding these limitations, our findings provided a strong foundation for the clinical translation of CLS as a promising dressing for anal fistula wounds. Looking forward to its industrialization feasibility, several aspects were noteworthy. Firstly, the CLS material was anticipated to be compatible with industrial sterilization methods such as gamma irradiation, which were unlikely to compromise its structural integrity and bioactivity [50]. Secondly, the cost-effectiveness of CLS was another attractive feature. The raw materials, derived from natural and abundant Calvatia lilacina spores. Coupled with a straightforward and scalable fabrication process, CLS held substantial potential to be an affordable and effective therapeutic option for anal fistula patients.

## Conclusion

In this paper, we have applied single-cell RNA sequencing to deeply explore that CLS reduced the number of monocytes to decrease the inflammatory state of the wound, while increased the communication between IL6^+^macrophages and fibroblasts, thus accelerated the transition from the inflammatory phase to the proliferative phase of the wound. These novel results may also guide future research on TCM in anal fistula wounds. Meanwhile, these findings provided key information and guidance for further investigation on the clinical applications of CLS.

## Supplementary Information

Supplementary material 1.

## Data Availability

The datasets analyzed during this study are available from the corresponding author on reasonable request.

## Abbreviations

ACTR2: Actin-related protein 2
ACTR3: Actin-related protein 3
BSA: Bovine serum albumin
CLS: Calvatia lilacina spore
COL1A1: Collagen type I alpha 1
CXCL-1: C-X-C motif chemokine ligand 1
CXCL-8: C-X-C motif chemokine ligand 8
DDRTree: Discriminative dimensionality reduction tree
ECM: Extracellular matrix
FBS: Fetal bovine serum
GAGs: Glycosaminoglycans
Hacat: Human keratinocyte
HE: Hematoxylin and eosin
HPLC: High-performance liquid chromatography
IF: Immunofluorescence
IHC: Immunohistochemistry
IL-6: Interleukin 6
JAK2: Janus kinase 2
MPs: Mononuclear phagocyte system
MSF: Mouse skin fibroblasts
PMA: Phorbol 12-myristate 13-acetate
Q-TOF–MS/MS: Quadrupole time-of-flight mass spectrometry
SEM: Scanning electron microscope
STAT3: Signal transducer and activator of transcription 3
TCM: Traditional Chinese Medicine
THP-1: Tohoku Hospital Pediatrics-1
TNF-α: Tumor necrosis factor-alpha
U-MAP: Uniform manifold approximation and projection
UPLC-MS: Ultra performance liquid chromatography-mass spectrometry
UV: Ultraviolet
VEGF: Vascular endothelial growth factor
WASF3: Wiskott-Aldrich syndrome protein family member 3
