# Supplementary material for: Single-cell RNA sequencing reveals the therapeutic mechanism of Calvatia lilacina in promoting wound healing of anal fistula
Source: Chin Med. 2026 Jan 7;21:14. doi: 10.1186/s13020-025-01293-w (PMC12777321; doi:10.1186/s13020-025-01293-w)
Supplement: Supplementary file 1 — Supplementary material 1. [file 13020_2025_1293_MOESM1_ESM.docx]

**Supplementary results**

| Supplementary table 1 Summary of the contents of fatty acids in the Calvatia lilacina | | | | | | | | |
| --- | --- | --- | --- | --- | --- | --- | --- | --- |
|  | | Code | | contents | | | Fatty Acids /Calvatia lilacina (mg/g) | |
| Saturated fatty acids(SFA) | | C16:0 | | Methyl palm | | | 1.270 | |
|  |  | C18:0 | | Stearic acid methyl ester | | | 0.616 | |
|  |  | C24:0 | | Methyl tetracosanoate | | | 0.121 | |
| Monounsaturated fatty acids(MUFA) | | C18:1n-9c | | Methy1-9-oleic acid methyl ester | | | 3.050 | |
| Polyunsaturated fatty acids(PUFA) | | C18:2n-6c | | Linoleic acid methyl ester | | | 0.815 | |
| Supplementary table 2 Summary of the contents of amino acids in the alcohol extract of Calvatia lilacina (mean ± SD, n = 3) \| \| \|; ntents \| amino acids/Calvatia lilacina （mg/g） \| contents \| amino acids/Calvatia lilacina （mg/g）; Aspartate \| 0.387 \| Isoleucine \| 0.169; Threonine \| 0.393 \| Leucine \| 0.093; Serine \| 0.41 \| Tyrosine \| 0.46; Glutamate \| 0.573 \| Phenylalanine \| 0.272; Glycine \| 0.207 \| Histidine \| 0.185; Alanine \| 0.3 \| Lysine \| 0.498; Cysteine \| 1.465 \| Arginine \| 0.263; Valine \| 0.803 \| Proline \| 0.293; Methionine \| 0.16 \| \| | | | | | | | | |
| Supplementary table 3 Investigation of the micromeritics characteristics of Calvatia lilacina (mean ± SD, n = 3) | | | | | | | | |
| contents | loose density | | tap density | | Hausner Ratio | carr index | | sliding angle |
|  | 0.1512±0.008 | | 0.2160±0.005 | | 1.431±0.084 | 29.96±4.064 | | 37.95±5.725 |

*UV and BCA Detection of total steroids, total polyphenols, total polysaccharides, and total protein in* *Calvatia lilacina spore powder*

S1-4.The standard regression equation curve ototal steroids, total polyphenols, total polysaccharides, and protein

*UPLC-MS and HPLC detection of representative monomeric components ergosterol and ergosterone in Calvatia lilacina spore powder.*

S5. (A) UPLC-MS spectra of 10 μg/mL ergosterone reference substance and 20 μ g/mL ergosterol reference substance. (B) UPLC-MS image of 0.5 mg/mL Calvatia lilacina spore powder alcohol extract. 1) ergosterone total ion chromatogram 2) ergosterol total ion chromatogram 3) ergosterone mass spectrometry ion scan 4) ergosterol mass spectrometry ion scan.

S6.(A), (C) HPLC spectra of 10 mg/mL Calvatia lilacina spore powder ethanol extract. (C) HPLC spectra of 5 mg/mL ergosterol reference substance. (D) HPLC spectra of 5 mg/mL ergosterone reference substance.

*GC detection of fatty acid in Calvatia lilacina spore powder.*

S7.GC chromatograms of Calvatia lilacina spore powder. (A) reference standard mixture; (B) Calvatia lilacina spore powder.

*CLS increased the IL-6 positive cell in raw264.7 and increased the expression levels of IL-6 and CXCL-1*

S8 Flow cytometry and ELISA detection of CLS intervention in raw264.7. (A) Flow cytometry was used to determine the ratio of IL-6 positive cells in raw264.7 after extraction. (B) Quantitative analysis the rate of IL-6 positive macrophage. (C,D) Elisa detection of IL-6 and CXCL-1 in cell supernatant.

*CLS promoted the transformation of fibroblasts into myofibroblasts and increased the expression of ACTR2 and ACTR3*

S9 Pseudotime and functional analysis of different fibroblast populations. (A) Distribution of fibroblast on the developmental tree by clusters; (B,C) Boxplots of myofibroblasts, inflammatory signature scores colored by fibroblast subtypes; (D,E) Violin plot of ACTR2, ACTR3 distribution across the control and treatment groups.

*CLS regulated granulation tissue metabolic profiles in the anal fistula*

S10 Granulation tissue metabolic profiles in anal fistula. (A-C) CLS regulated key metabolic pathways (control group vs treatment group).
